# L-arginine dependence of breast cancer – molecular subtypes matter.

**DOI:** 10.1186/s12885-025-13908-4

**Published:** 2025-03-26

**Authors:** Juliane Hannemann, Leticia Oliveira-Ferrer, Anne Kathrin Goele, Yoana Mileva, Fiona Kleinsang, Antonia Röglin, Isabell Witzel, Volkmar Müller, Rainer Böger

**Affiliations:** 1 Institute of Clinical Pharmacology and Toxicology, University Medical Center Hamburg-Eppendorf, Hamburg, Germany; 2 Department of Gynecology, University Medical Center Hamburg-Eppendorf, Hamburg, Germany; 3 Department of Gynecology, University Hospital Zürich, Zürich, Switzerland

**Keywords:** Breast cancer subtypes, Biomarkers, Cancer metabolism, Breast cancer cell lines, Arginase, Translational research, Prospective cohort, Mortality

## Abstract

L-arginine limits proliferation in highly proliferative tissues. It is a substrate for nitric oxide synthases, arginases; its methylation by protein-L-arginine methyltransferases (PRMTs) leads to asymmetric (ADMA) and symmetric dimethylarginine (SDMA). We measured L-arginine and its metabolites L-ornithine, L-citrulline, ADMA, and SDMA in a prospective cohort of 243 women with primary breast cancer (BC) and their associations with mortality and disease recurrence during 88 (IQR, 82–93) months of follow-up. We quantified these metabolites and expression of genes involved in L-arginine metabolic pathways in MCF-7, BT-474, SK-BR-3, MDA-MB-231, and MDA-MB-468 cells representing ER-positive, HER2-positive, and triple-negative BC compared to MCF-12 A cells. Plasma L-arginine and ADMA concentrations were elevated in 47 patients with recurrent disease and in 34 non-survivors. ADMA was significantly associated with mortality and recurrent disease in Luminal A patients; low L-citrulline was significantly associated with survival in triple-negative BC. In all BC cells except MCF-7, *DDAH1* and *DDAH2* expression was higher than in MCF-12 A (*DDAH1*: 32–44 fold, *DDAH2*: 1.7–4.2 fold; *p* < 0.05). By contrast, MCF-7 cells showed low *DDAH1* and *DDAH2*, but high *PRMT4* and *PRMT6* expression and high L-arginine content. BT-474 and MDA-MB-468 cells showed high *ARG2* expression and high L-ornithine concentrations, and MDA-MB-468 cells had the highest L-citrulline/L-arginine ratio. In conclusion, regulation of L-arginine metabolic pathways shows a complex and differential pattern between BC subtypes. ADMA is a prognostic biomarker in Luminal A patients; its metabolizing enzyme, DDAH, is highly overexpressed in BC cells. Thus, fingerprinting of L-arginine metabolism may offer novel personalized treatment options within BC subtypes.

**Supplementary Information:**

The online version contains supplementary material available at 10.1186/s12885-025-13908-4.

## Introduction

Breast cancer is the leading malignancy in women worldwide. With an estimated 2.3 million newly diagnosed cases and almost 700.000 deaths annually, it accounts for 12% of all newly diagnosed cancer cases and 7% of all cancer-related deaths, respectively [1]. During the last decades, our understanding of the biology of breast cancer as a heterogeneous disease consisting of clinically distinct subtypes has evolved. Four subtypes of breast cancer, identified by gene expression profiling and first described by Perou et al. [2] in 2000, have been further characterized by many research groups since then [3–5] and linked to therapy response [6, 7]. These subtypes (Luminal A, Luminal B, HER2-positive, and triple-negative) significantly differ in terms of incidence, prognosis, treatment options, and treatment response [8]. However, the current stratification based on a combination of clinical parameters and the histopathological markers estrogen receptor (ER), progesterone receptor (PR), and HER2 (human epidermal growth factor receptor-2) might not completely mirror the complexity of the disease. Mortality rates are still high and some tumors show therapy resistance whilst others do not. Therefore, novel biomarkers guiding therapy decisions are still urgently needed.

Metabolic reprogramming has become an emerging hallmark of cancer [9]. Therefore, there is increasing research interest in cell intrinsic metabolic preferences in cancer and its therapeutic exploitation in different cancer entities [10, 11]. The semi-essential, proteinogenic amino acid L-arginine has been suggested to act as a cell growth-limiting nutrient, ever since L-arginine availability was shown, first in neonatal rats [12], to be critical in situations of accelerated cell growth. It is assumed that tumor cells are auxotrophic for L-arginine, and L-arginine deprivation will lead to metabolite deprivation, decreased DNA synthesis, and finally to apoptosis of these cells [13]. Therefore, Larginine deprivation has been proposed as an attractive approach for cancer therapy (reviewed in [14]). However, the role of L-arginine in breast cancer has remained controversial: Early studies showed higher proliferation rates in breast cancer cells in patients undergoing L-arginine supplementation as compared to patients without Larginine supplementation [15]. Recent results show that dietary L-arginine significantly inhibited tumor growth and prolonged survival in a mouse model [16]. These differences may relate to differences in intrinsic subtypes of breast cancer studied, as it is well known that hormone-receptor-(ER, PR)-positive, HER2-positive, and triple-negative breast cancers show major differences in metabolic demands [17, 18].

L-arginine is involved in various enzymatic pathways, as depicted in Supplementary Fig. 1. It can be converted to L-ornithine by arginases 1 and 2, it can be metabolized to L-citrulline and nitric oxide (NO) by NO synthases, and – when present as part of a peptide chain – it can be mono- or dimethylated by a family of enzymes named protein-L-arginine methyltransferases (PRMTs), resulting in the formation of monomethyl-L-arginine, asymmetric (ADMA) or symmetric dimethylarginine (SDMA) (for review, cf. [19]). We aimed to analyze L-arginine and its major metabolites in the plasma of patients with breast cancer and relate these metabolite levels to clinical outcome of the patients. As metabolic dysregulation occurring in breast cancer cells affects only a small percentage of the body’s total cells, metabolite measurements may show only minimal differences despite major metabolic changes in the cancer cells themselves. However, metabolic analysis of native tumor tissues is not always readily available; therefore, we performed additional molecular studies with breast cancer cell lines in-vitro. For this, we selected breast cancer cell lines representing the molecular subtypes of breast cancer to assess intracellular metabolite levels and gene expression profiles of L-arginine-metabolizing enzymes and compared them to a cell line representing healthy breast tissue.

## Patients and methods

### Patients and study protocol

We recruited 271 women who presented with a diagnosis of breast cancer at the breast cancer center of the University Medical Center Hamburg-Eppendorf between July, 2010, and August, 2013. Supplementary Fig. 2 displays the flow diagram of patients in this study; 27 patients were excluded because they had recurrent disease, and one patient was excluded because of missing biosamples; a final number of 243 women with primary breast cancer and samples available for biomarker measurement were included in our analyses. All patients had given their informed consent to include their routine blood samples into the local biobank and their inclusion in biomarker analyses. The study protocol had been approved by the Ethics Committee of the Board of Physicians of Hamburg (OB/V/03).

Follow-up during in-hospital treatment was performed based on hospital records. Most patients remained under regular out-patient supervision in the University breast center for clinical follow-up. In addition, and for those under clinical continuation treatment elsewhere, we performed telephone interviews using a structured questionnaire. Repeated phone calls were initiated to ensure the highest possible follow-up rate. The questionnaire developed for and used in this study is available in Supplementary Materials to this article.

In addition, we collected blood samples from 129 healthy female blood donors who had consented to donate a blood sample for research purposes according to an approval by the Ethics Committee of the Board of Physicians of Hamburg (2022-300225-WF).

### Biochemical analyses

L-arginine, L-citrulline, L-ornithine, ADMA, and SDMA were quantified in EDTA plasma samples and in breast cancer cell lysates. For this, cell culture medium was aspirated from confluent cell culture dishes and discarded, cells were washed once with ice-cold PBS, scraped into microcentrifuge vials, lysed by repeated freeze-thawing followed by ultrasound bath (6 min), and resuspended in a total volume of 100 μL with PBS. 10 μL were used for protein measurement using a nanophotometer N60 (Implen GmbH, Munich, Germany). For analysis of L-arginine and related metabolites by ultra-performance liquid chromatography-tandem mass spectrometry (UPLC-MS/MS), duplicate aliquots of 25 μL of plasma or cell lysates were diluted in 100 μL methanol to which stable isotope-labelled internal standards had been added. Subsequently, the compounds were converted into their butyl ester derivatives as described elsewhere [20]. Quantification of analytes was performed on a Waters UPLC-MS/MS platform (Xevo TQ-S cronos, Waters GmbH, Eschborn, Germany) applying an ACQUITY UPLC BEH C_18_ column (2.1 × 50 mm, 1.7 μm, Waters GmbH) for chromatographic separation. The coefficient of variation for the quality control samples was below 6% for all compounds.

### Cell culture

We utilized six different cell lines in this study; their relation to the subtypes of breast cancer is listed in Table 1. All cell lines (MCF-12A, MCF-7, BT-474, SK-BR-3, MDA-MB-468, MDA-MB-231) were obtained from ATCC (American Tissue Culture Collection). With the exception of MCF-12A cells, all cell lines were cultured in RPMI1640 supplemented with 10% FCS (Capricorn Scientific, Ebsdorfergrund, Germany) and a final concentration of 100 U/mL penicillin, 100 μg/mL streptomycin (Merck, Darmstadt, Germany). MCF-12A cells were cultured in DMEM/F12 medium supplemented with 5% horse serum, 100 U/mL penicillin, 100 μg/mL streptomycin, 100 ng/mL cholera toxin, 20 ng/mL human EGF, 10 μg/mL insulin, and 500 ng/mL hydrocortisone. All cell lines were grown at 37 °C and 5% CO_2_. Subculturing was performed according to the suppliers’ recommendations.

**Table 1:** Normal breast and breast cancer cell lines used in this study

Cell line	Representative of subtype	Immunoprofile
MCF-12 A	Normal	ER^+^, PR^+^, HER2^−^
MCF-7	Luminal A	ER^+^, PR^+^, HER2^−^
BT-474	Luminal B	ER^+^, PR^+^, HER2^+^
SK-BR-3	HER2-positive	ER^−^, PR^−^, HER2^+^
MDA-MB-468 MDA-MB-231	Triple-negative (basal-like) Triple-negative (claudin-low)	ER^−^, PR^−^, HER2^−^ ER^−^, PR^−^, HER2^−^

Abbreviations: ER, estrogen receptor; PR, progesterone receptor; HER2, human epidermal growth factor receptor-2

### Assessment of gene expression in cultured human breast cancer cell lines

Cells grown to 80–90% confluence were lysed in 1 ml ice cold Trizol (ThermoFisher, Waltham, MA, USA) and further processed with the PureLink™ RNA Mini Kit and PureLink™ DNase (both Thermofisher) according to the manufacturer’s instruction. gDNA digestion and RNA integrity were checked by agarose gel electrophoresis and RNA was stored at -80 °C until further use. 2.5 μg of each RNA samples was reverse transcribed by SuperScript IV VILO™ (ThermoFisher) following strictly the manufacturer’s instruction.

Quantitative real-time PCR (qRT-PCR) was performed using 12.5 ng cDNA, Taqman Fast Advanced Master Mix, and gene-specific Taqman™ assays in a volume of 10 μl according to the manufacturer’s instruction. Assays used for the genes of interest contained unlabeled gene-specific primers and a 5’-FAM Taqman™ MGB probe with a 3’-nonfluorescent quencher. The specific TaqMan qRT-PCR assays (all ThermoFisher) used in this study are listed in Supplementary Table 1.

A 5’-VIC-labeled assay for *ACTB* (Hs01060665_g1, Thermofisher) was used as reference gene. Non-template controls were included for each assay; all samples were run as technical triplicates on a Quantstudio 5 System (ThermoFisher). UNG incubation for 2 min at 50 °C, and activation for 10 min at 95 °C, 40 cycles of denaturation (15 s at 95 °C) and annealing/extension (1 min at 60 °C). Relative gene expression was subsequently determined using the ΔΔCt method [21].

### Calculations and statistical analyses

From biomarker concentrations measured, we calculated the L-arginine/ADMA ratio, a measure for NO synthase substrate availability, as well as the L-citrulline/L-arginine ratio, a surrogate measure of NOS catalytic activity, and the L-ornithine/L-arginine ratio, a measure of the arginase catalytic activity.

Data are presented as mean ± standard deviation for all continuous variables, and as number of observations with percentage given in brackets for categorical variables. Differences between groups were tested by one-way ANOVA. The χ^2^ test was used for comparison of categorical variables between groups. Survival analyses were performed using Kaplan–Meier curves comparing patients with ADMA and SDMA above or below the cut-off value determined in receiver-operated curve (ROC) analyses. The Youden index was calculated to identify the optimal cut-off for biomarkers [22]. Linear regression analysis was performed to assess the correlation of L-arginine metabolites with age. Hazard ratios (HR) and 95% confidence intervals (CI) were calculated by multivariable-adjusted logistic regression analyses including age and a co-variable. All statistical analyses were performed using SPSS (version 25; IBM Corporation, Armonk, NY, USA) and GraphPad Prism (version 6.01, GraphPad Software, San Diego, CA, USA). For all tests, *p* < 0.05 was considered statistically significant.

## Results

### Baseline characteristics

We analysed blood samples drawn before surgery and initiation of systemic therapy from 271 women diagnosed with breast cancer in the University Medical Center Hamburg-Eppendorf. 27 women were excluded from our analysis because they had recurrent disease; all other women had primary breast cancer. L-Arginine-related metabolite concentrations were not available in one woman, so that our final cohort comprised 243 women with primary breast cancer, for whom complete follow-up data were collected during a median of 88 (IQR, 82–93) months. Supplementary Fig. 2 shows the CONSORT flow diagram of our study. The control group comprised 129 healthy female blood donors; the mean age of this group was 51.6 ± 10.8 years. Patients in our cohort had a mean age of 60.2 ± 13.2 years.

Histologically confirmed data on intrinsic subtypes were not available for 12 women; thus, subgroup analyses were performed for 231 patients. Out of these, 111 women (45.7%) had Luminal A breast cancer, 67 (27.6%) had Luminal B, 17 (7.0%) had HER2-positive breast cancer, and 36 women (14.9%) were triple-negative. 53% had a Ki67 greater than or equal to 20%. Beyond primary surgery, oncological treatment comprised radiotherapy in 80.2% of the patients, chemotherapy in 39.5%, and endocrine therapy in 56.0%. Overall mortality in our cohort was 14.0%; mortality was lowest in Luminal A and Luminal B breast cancer (12.6% and 13.4%, respectively), and higher in HER2-positive and triple-negative breast cancer (17.6% and 22.2%, respectively). The baseline characteristics of our cohort are presented in Table 2.

**Table 2:** Baseline characteristics of the breast cancer patient cohort

	Total cohort	Deceased	Survived	*p*	Recurrence	No recurrence	*p*
**Demographics/Anthropometrics**							
*N*	243	34 (14.0)	209 (86.0)	–	47 (19.3)	196 (80.7)	–
Age (years)	60.2 ± 13.2	58.7 ± 15.1	60.4 ± 12.9	n.s.	56.6 ± 15.2	61.0 ± 12.6	**0.040**
Weight (kg)	71.8 ± 13.9	75.0 ± 18.7	71.4 ± 13.0	n.s.	70.3 ± 14.1	72.2 ± 13.9	n.s.
Height (cm)	165.8 ± 6.9	164.6 ± 5.6	166.0 ± 7.1	n.s.	164.6 ± 5.7	166.1 ± 7.2	n.s.
BMI	26.2 ± 5.2	27.7 ± 7.2	25.4 ± 4.8	n.s.	26.0 ± 5.4	26.2 ± 5.1	n.s.
Family history	133	17 (12.8)	116 (87.2)	n.s.	31 (23.3)	102 (76.7)	n.s.
History of other malignancies	45	8 (17.8)	37 (82.2)	n.s.	14 (31.1)	31 (68.9)	**0.026**
**Histological Subtype**							
IDC	181	29 (16.0)	152 (84.0)	n.s.	36 (19.9)	145 (90.1)	n.s.
ILC	30	3 (10.0)	27 (90.0)		5 (6.7)	25 (83.3)	
DCIS / LCIS	21	1 (4.8)	20 (95.2)		6 (28.6)	15 (71.4)	
Other	11	1 (9.1)	10 (88.9)		0	11 (100.0)	
**Tumor staging**							
T1	138	17	121	n.s.	23	115	n.s.
T2	66	13	53		15	51	
T3	9	2	7		2	7	
T4+	5	1	4		2	3	
Tis	21	1	18		5	14	
T not specified	4	0	6		0	4	
N0	145	18	127	n.s.	21	124	n.s.
N1	55	8	47		12	43	
N2	18	4	14		4	14	
N not specified	25	4	21		10	15	
M0	83	16	67	n.s.	20	63	n.s.
M1	5	2	3		2	3	
M not specified	155	16	139		25	130	
Lymphatic invasion	43	9	34	n.s.	11	32	n.s.
Vascular invasion	3	1	2	n.s.	1	2	n.s.
**Breast cancer subtypes and proliferation marker status**							
Luminal A	111 (45.7)	14 (12.6)	97 (87.4)	n.s.	12 (10.8)	99 (89.2)	**0.009**
Luminal B	67 (27.6)	9 (13.4)	58 (86.6)		19 (28.4)	48 (71.6)	
HER2-positive	17 (7.0)	3 (17.6)	14 (82.4)		2 (11.8)	15 (88.8)	
Triple-negative	36 (14.9)	8 (22.2)	28 (77.8)		11 (30.6)	25 (69.4)	
Ki67 ≥ 20%	103 (42.4)	18 (17.5)	85 (82.5)	n.s.	26 (25.2)	77 (74.8)	**0.013**
**Oncological treatment in addition to surgery**							
Endocrine therapy	136 (56.0)	20 (58.8)	116 (55.5)	n.s.	23 (50.0)	113 (57.7)	n.s.
Chemotherapy	96 (39.5)	17 (50.0)	79 (37.8)	n.s.	24 (52.2)	72 (36.7)	n.s.
Radiotherapy	195 (80.2)	26 (76.5)	169 (80.9)	n.s.	33 (70.2)	162 (82.7)	**0.034**

Numbers are given as mean ± standard deviation for continuous variables and as N (per cent) for categorical variables; for tumor staging only N is given for clarity of reading. Percentages add up horizontally to indicate the proportion of surviving vs. deceased patients and patients with or without recurrent disease, respectively, in each line. Abbreviations: BMI, body mass index; DCIS, ductal carcinoma in situ; ER, estrogen receptor; HER2-positive, human epidermal growth factor receptor-2-positive breast cancer; IDC, invasive ductal carcinoma; ILC, invasive lobular carcinoma; Ki67, percentage of Ki67 proliferation marker-positive cells within the tumor; LCIS, lobular carcinoma in situ; PR, progesterone receptor. * *P* < 0.05 for deceased vs. surviving patients and for patients with recurrent breast cancer versus those without recurrent disease

### Concentrations of L-arginine-related metabolites in women with breast cancer

In a statistical model including age as a co-variate, the plasma concentrations of L-arginine and L-citrulline were not significantly different between patients and controls; however, L-ornithine concentration showed a trend to be higher in patients than in controls (Table 3). In consequence, the L-ornithine/L-arginine ratio was significantly elevated in breast cancer patients versus controls. The plasma concentrations of ADMA and SDMA concentrations were not significantly different between patients and controls.

**Table 3:** Concentrations of L-arginine-related biomarkers in plasma of breast cancer patients and healthy controls

	Breast cancer patients	Controls	*p*	Breast cancer patients			Breast cancer patients		
				Deceased	Survived	*p*	Recurrence	No recurrence	*p*
L-Arginine	64.9 ± 26.5	102.5 ± 27.4	n.s.	73.4 ± 36.9	63.5 ± 24.2	**0.043**	74.0 ± 35.7	62.7 ± 23.3	**0.009**
L-Citrulline	32.5 ± 10.2	35.3 ± 9.6	n.s.	33.5 ± 11.2	32.3 ± 10.0	n.s.	32.6 ± 10.7	32.4 ± 10.1	n.s.
L-Ornithine	78.1 ± 25.2	66.6 ± 17.9	0.068	81.8 ± 29.7	77.5 ± 24.4	n.s.	78.1 ± 27.3	78.1 ± 24.7	n.s.
ADMA	0.483 ± 0.103	0.504 ± 0.096	n.s.	0.526 ± 0.122	0.476 ± 0.098	**0.008**	0.499 ± 0.133	0.479 ± 0.094	n.s.
SDMA	0.456 ± 0.135	0.501 ± 0.098	n.s.	0.461 ± 0.147	0.455 ± 0.133	n.s.	0.448 ± 0.146	0.457 ± 0.133	n.s.
Cit/Arg Ratio	0.56 ± 0.28	0.36 ± 0.10	n.s.	0.53 ± 0.25	0.57 ± 0.29	n.s.	0.50 ± 0.22	0.58 ± 0.29	n.s.
Orn/Arg Ratio	1.42 ± 0.97	0.67 ± 0.18	**0.046**	1.28 ± 0.71	1.44 ± 1.01	n.s.	1.25 ± 0.80	1.46 ± 1.01	n.s.
Arg/ADMA Ratio	136.1 ± 47.3	207.0 ± 54.6	n.s.	140.3 ± 56.6	135.5 ± 45.7	n.s.	148.3 ± 53.5	133.2 ± 45.3	0.050

Data are given as mean ± standard deviation. Biomarker concentrations are in μmol/L; biomarker ratios are without dimension. *P* values were calculated by ANOVA followed by Dunn’s multiple comparisons test. Statistical differences between groups were calculated by unpaired, two-sided Mann-Whitney test. Abbreviations: ADMA, asymmetric dimethylarginine; Arg/ADMA Ratio, ratio of L-arginine over ADMA; Cit/Arg Ratio, ratio of L-citrulline over L-arginine; Orn/Arg Ratio, ratio of L-ornithine over L-arginine; SDMA, symmetric dimethylarginine

**Fig. 1:**
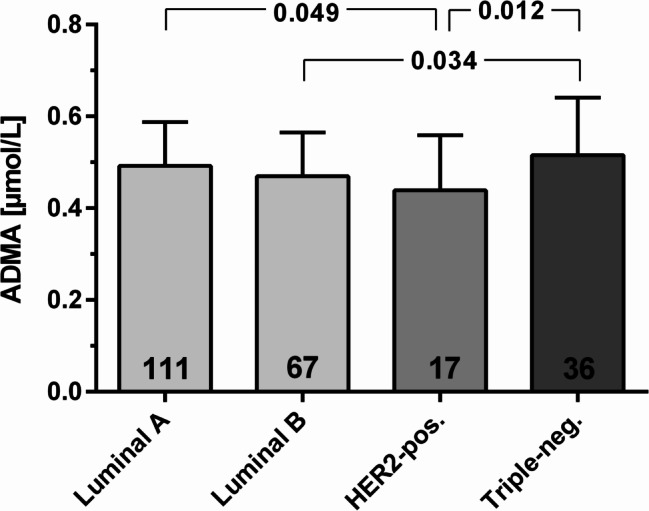
Plasma concentrations of ADMA in patients with breast cancer according to subtypes. Data are mean ± standard deviation. The number of patients in each subtype is indicated within the columns. Abbreviations: ADMA, asymmetric dimethylarginine. HER2+, human epidermal growth factor receptor-2-positive breast cancer. The *p* values for differences between subtypes were derived from one-way ANOVA

However, we found a significantly different concentration of mean ADMA between subtypes of breast cancer patients (*p for trend* = 0.040; Fig. 1); namely patients with triple-negative cancer had the highest mean ADMA concentration, whilst mean ADMA was lowest in HER2-positive patients. The plasma concentrations of all other L-arginine-related biomarkers showed no significant differences between intrinsic subtypes of breast cancer (Table 4).

**Table 4:** Concentrations of L-arginine-related biomarkers in plasma and clinical characteristics in breast cancer subtypes

	Luminal A	Luminal B	HER2-positive	Triple-negative	*p* for trend
**N**	111	67	17	36	
**Biomarker concentrations (μmol/L)**					
L-Arginine	66.4 ± 24.7	60.6 ± 23.2	62.2 ± 28.6	71.5 ± 36.9	n.s.
L-Citrulline	32.9 ± 8.8	33.0 ± 11.4	32.5 ± 14.2	30.6 ± 9.4	n.s.
L-Ornithine	80.8 ± 23.6	75.2 ± 20.8	79.2 ± 46.6	76.5 ± 26.4	n.s.
ADMA	0.492 ± 0.096	0.470 ± 0.095	0.439 ± 0.120	0.516 ± 0.125	**0.040**
SDMA	0.461 ± 0.120	0.447 ± 0.157	0.440 ± 0.121	0.479 ± 0.157	n.s.
Cit/Arg Ratio	0.57 ± 0.33	0.59 ± 0.23	0.56 ± 0.27	0.50 ± 0.20	n.s.
Orn/Arg Ratio	1.46 ± 1.08	1.46 ± 1.04	1.41 ± 0.88	1.25 ± 0.62	n.s.
Arg/ADMA Ratio	135.9 ± 43.0	131.3 ± 45.1	143.1 ± 49.1	141.6 ± 63.9	n.s.
**Clinical parameters**					
Age	62.5 ± 11.3	56.7 ± 15.9	60.3 ± 15.2	59.0 ± 14.2	**0.037**
Radiotherapy	91 (82.0)	54 (80.6)	12 (70.6)	29 (80.6)	n.s.
Chemotherapy	13 (11.7)	42 (62.7)	14 (82.4)	26 (72.2)	**< 0.001**
Endocrine Therapy	80 (72.1)	48 (71.6)	3 (17.6)	3 (8.3)	**< 0.001**
Deceased	14 (12.6)	9 (13.4)	3 (17.6)	8 (22.2)	n.s.
Recurrent cancer	11 (9.9)	19 (28.4)	2 (11.8)	11 (30.6)	**0.004**

Data are mean ± standard deviation for continuous variables and N (per cent) for categorical variables. Abbreviations: ADMA, asymmetric dimethylarginine; Arg/ADMA Ratio, ratio of L-arginine over ADMA; Cit/Arg Ratio, ratio of L-citrulline over L-arginine; Orn/Arg Ratio, ratio of L-ornithine over L-arginine; SDMA, symmetric dimethylarginine

### Association of L-arginine and its metabolites with clinical outcome in breast cancer patients

We next assessed the association of L-arginine-related metabolites with disease recurrence and total mortality during follow-up using ROC analysis. After a median follow-up of 88 (IQR, 82–93) months, 47 patients had developed recurrent disease and 34 patients had died. For the total cohort, patients who deceased had significantly higher L-arginine and ADMA plasma concentrations than survivors (Table 3). Higher L-arginine concentration in plasma was also significantly associated with disease recurrence during follow-up, whilst none of the other L-arginine-related metabolites showed a significant association with mortality or disease recurrence (Table 3).

**Fig. 2:**
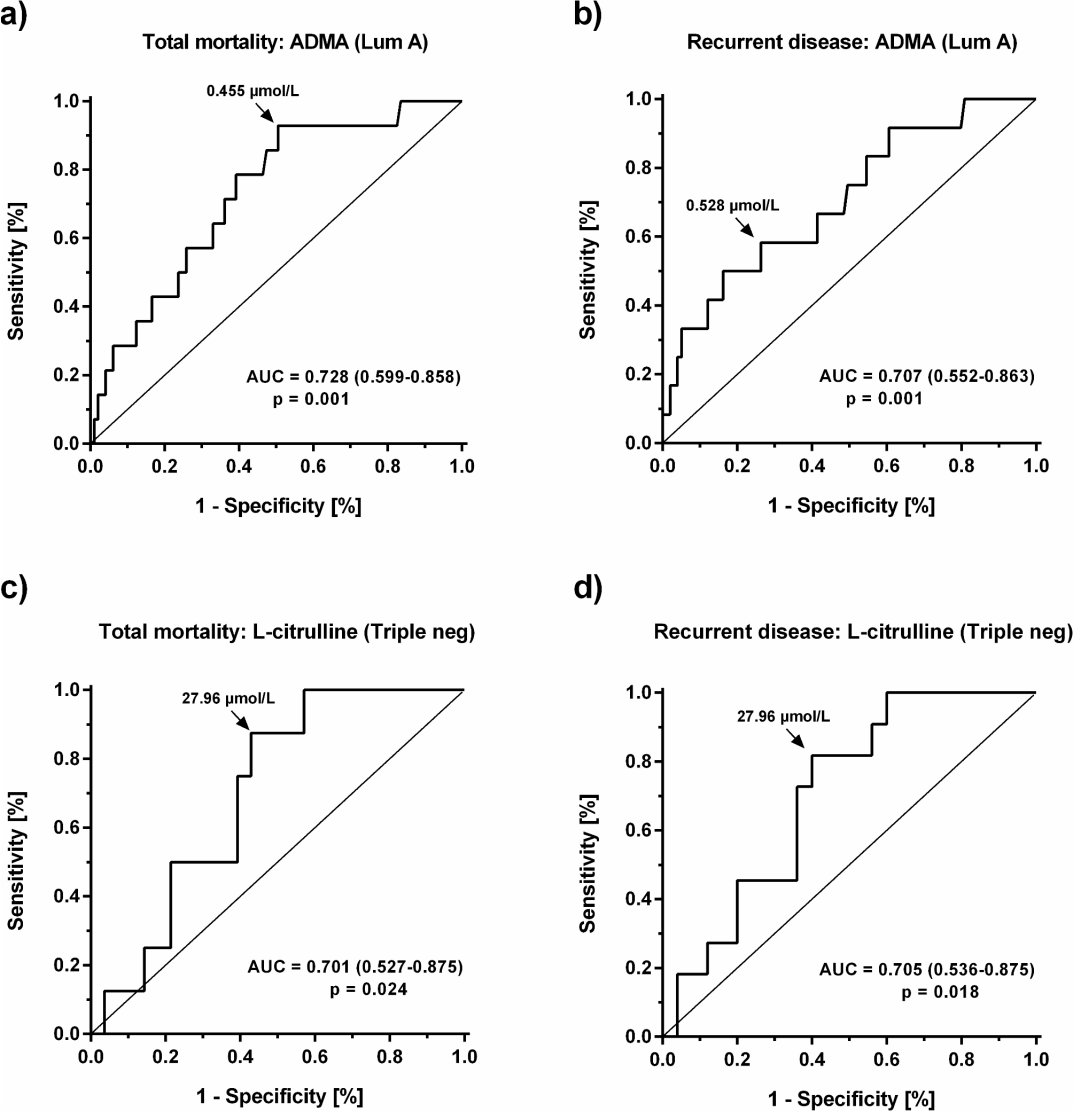
Receiver-operated curve (ROC) analysis of biomarkers related to mortality and recurrence of breast cancer. **a**) ADMA and total mortality in patients with Luminal A breast cancer; **b**) ADMA and recurrent disease in patients with Luminal A breast cancer; **c**) L-citrulline and total mortality in patients with triple-negative breast cancer; **d**) L-citrulline and recurrent disease in patients with triple-negative breast cancer. The arrows point to the optimal cut-off values to differentiate between survivors and non-survivors (a and c) and patients with or without recurrent disease (b and d)

Amongst the subtypes of breast cancer, we found statistically significant associations of ADMA with total mortality and with recurrent disease in patients with Luminal A breast cancer (Fig. 2a and b), and significant associations of L-citrulline concentration with mortality and recurrent disease in patients with triple-negative breast cancer (Fig. 2c and d). ROC analyses for total mortality in all subtypes are presented in Supplementary Fig. 3 to 7.

**Fig. 3:**
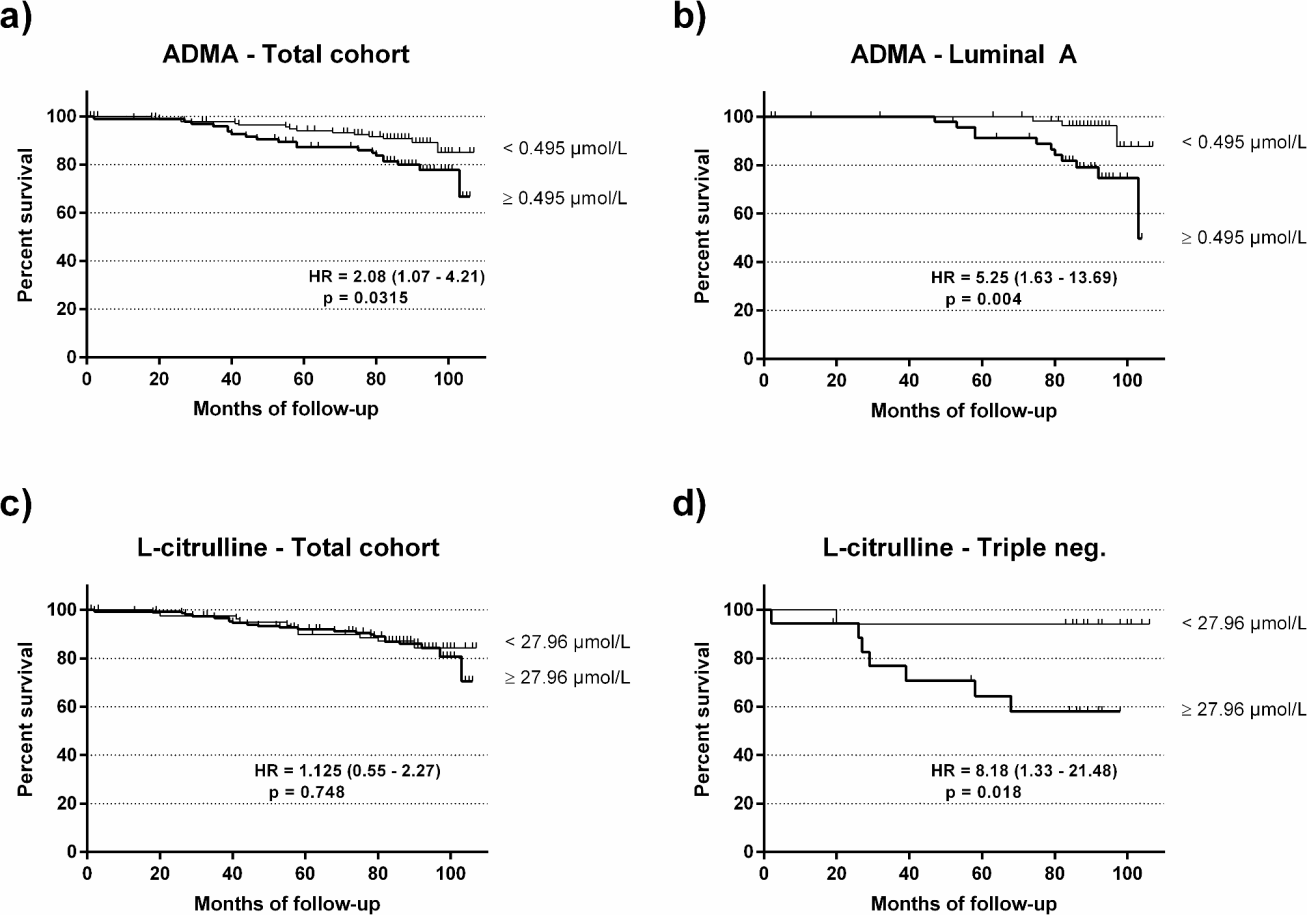
Kaplan-Meier survival analyses for total mortality in breast cancer patients. (**a**) Total mortality in patients with breast cancer (total cohort) with high or low ADMA plasma concentration; (**b**) total mortality in patients with Luminal A breast cancer with high or low ADMA plasma concentration; (**c**) total mortality in patients with breast cancer (total cohort) with high or low L-citrulline plasma concentration; (**d**) total mortality in patients with triple-negative breast cancer with high or low L-citrulline plasma concentration

We next constructed Kaplan Meier survival curves after splitting the patient population according to the optimal cut-off biomarker concentrations assessed in ROC analyses. We found that ADMA concentrations ≥ 0.495 μmol/L were significantly associated with increased mortality, both in the total breast cancer patient cohort (HR = 2.08 (1.07–4.21), *p* = 0.0315; Fig. 3a) and in the subgroup of patients with Luminal A breast cancer (HR = 5.25 (1.63–13.69); *N* = 111; *p* = 0.004; Fig. 3b). L-citrulline concentrations above 27.96 μmol/L were not significantly associated with mortality in the total cohort (Fig. 3c), but we found a highly significant association between high L-citrulline concentration and shorter survival time in patients with triple-negative breast cancer (HR = 8.18 (1.33–21.48); *N* = 36; *p* = 0.018; Fig. 3d).

### Concentrations of L-arginine and related metabolites in breast cancer cell lines in-vitro

**Fig. 4:**
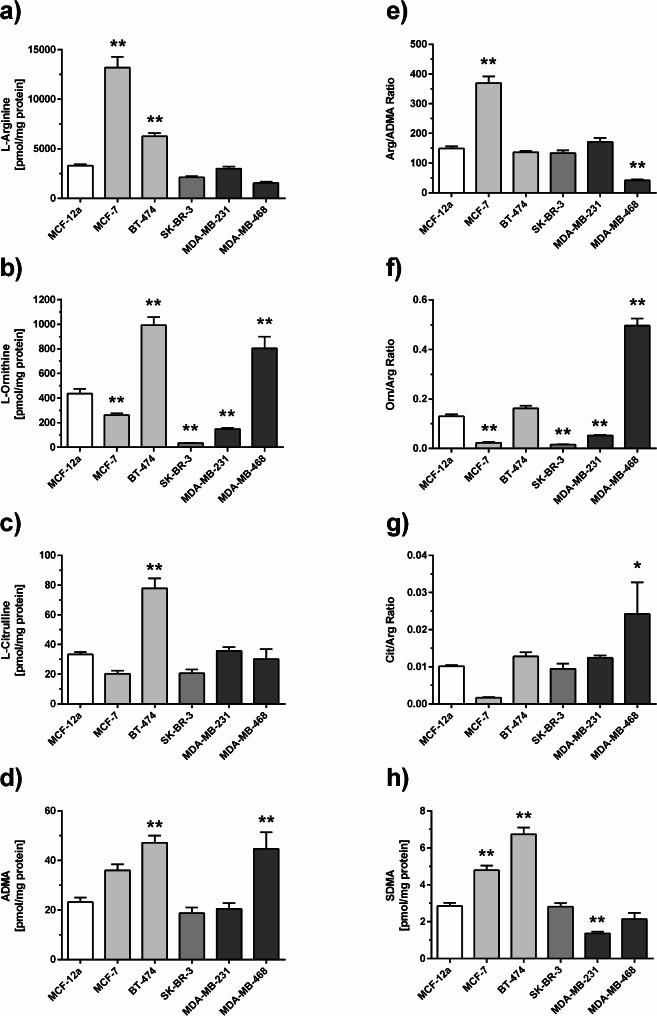
Intracellular concentrations of L-arginine-related metabolites in subtype-specific cell lines in-vitro. Data show concentrations of (**a**) L-arginine, (**b**) L-ornithine, (**c**) L-citrulline, (**d**) ADMA, (**e**) the Larginine/ADMA ratio, (**f**) the L-ornithine/L-arginine ratio, (**g**) the L-citrulline/L-arginine ratio, and (**h**) SDMA. The insert in chart h) displays an enlarged representation of the data. * *p* < 0.05, ** *p* < 0.001 as compared to MCF-12 A normal breast epithelial cells in one-way ANOVA followed by Dunnett’s multiple comparisons test. Differences in color shading of the bars indicate different intrinsic subtypes of breast cancer. Please note the different scaling of the y-axis in figures d) and h)

The intracellular concentrations of L-arginine and its metabolites varied greatly amongst cell lines representing different breast cancer subtypes: by comparison with MCF-12A breast epithelial cells, MCF-7 cells and BT-474 cells had significantly and highly elevated L-arginine concentrations (Fig. 4a). L-Ornithine concentrations varied amongst cell lines, with significantly elevated L-ornithine/L-arginine ratio in MDA-MB-468 cells (Fig. 4b and f). L-Citrulline concentration was significantly elevated in BT-474 cells but with no significant elevation of the L-citrulline/L-arginine ratio; by contrast, this ratio was significantly elevated in MDA-MB-468 cells (Fig. 4c and g).

ADMA concentration was significantly higher in BT-474 cells and in MDA-MB-468 cells as compared to MCF-12A cells (Fig. 4d). The L-arginine/ADMA ratio was significantly higher in MCF-7 and significantly lower in MDA-MB-468 cells as compared to MCF-12A cells (Fig. 4e). Overall, SDMA concentration was about one order of magnitude lower than that of ADMA in all cell lines; however, the two hormone-receptor (ER, PR)-positive cell lines, MCF-7 and BT-474, had significantly higher SDMA levels than MCF-12A cells (Fig. 4h).

### Expression of genes determining L-arginine metabolism in breast cancer cell lines in-vitro

**Fig. 5:**
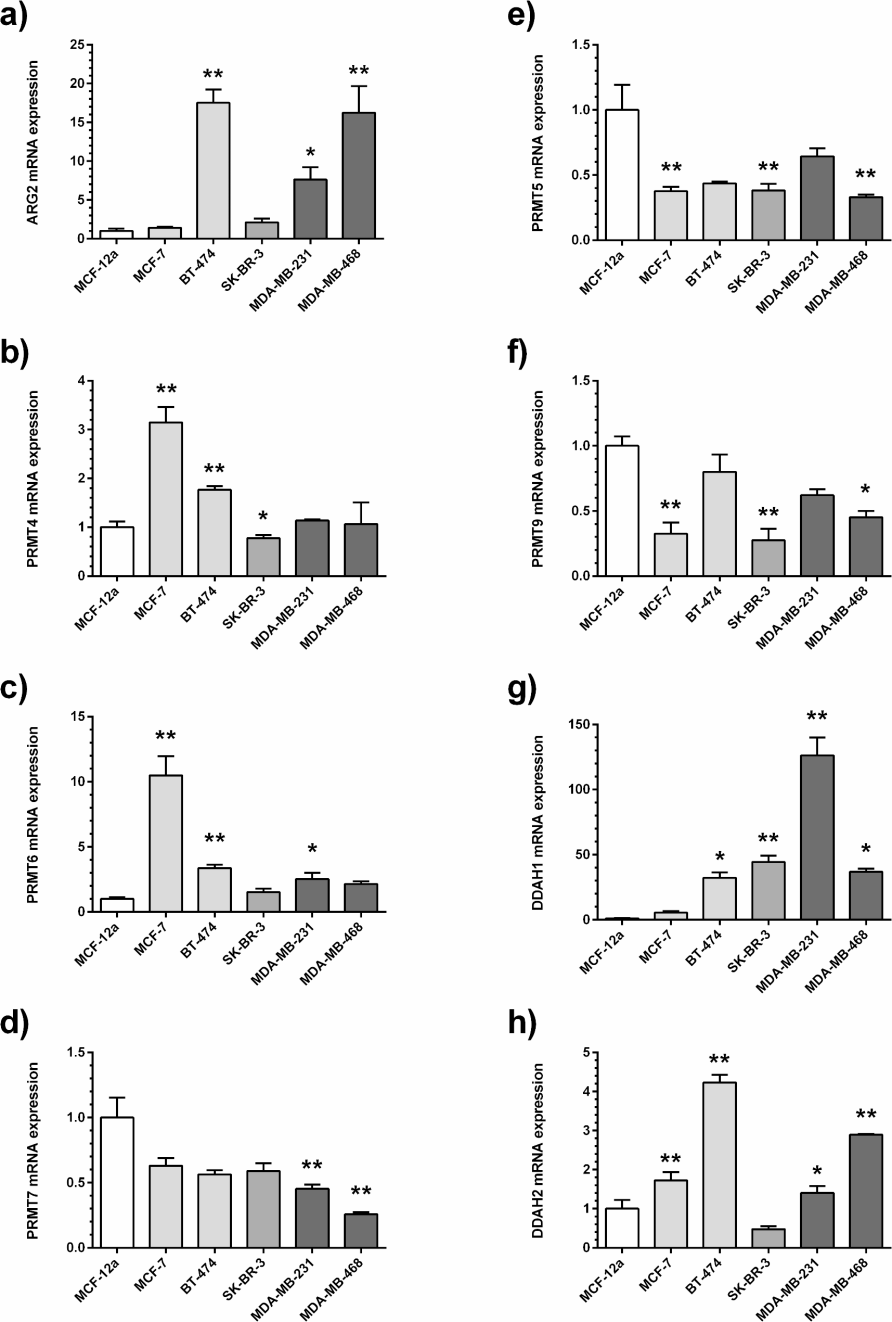
Gene expression measured by quantitative real-time RT-qPCR for major genes involved in L-arginine-metabolizing pathways: (**a**) arginase-2 (ARG2), (**b**) PRMT4, (**c**) PRMT6, (**d**) PRMT7, (**e**) PRMT5, (**f**) PRMT9, (**g**) DDAH1, (**h**) DDAH2. * *p* < 0.05, ** *p* < 0.001 as compared to MCF-12 A normal breast epithelial cells in one-way ANOVA followed by Dunnett’s multiple comparisons test. Differences in colour shading of the bars indicate different intrinsic subtypes of breast cancer

Whilst *ARG1* was not expressed, *ARG2* mRNA was found in all breast cancer cell lines. Its expression was highest in BT-474, MDA-MD-231, and MDA-MB-468 cells (Fig. 5a). *NOS I*, *NOS II*, and *NOS III* showed minimal expression levels in all cell lines (data not shown).

Amongst genes involved in dimethylarginine biosynthesis and metabolism, *PRMT4* and *PRMT6* gene expression was upregulated in the ER-positive cell lines MCF-7 and BT-474 (Fig. 5b and c), whilst *PRMT5* and *PRMT9* were downregulated in MCF-7 and MDA-MB-468 cells (Fig. 5e and f). *PRMT7* was downregulated in both triple-negative cell lines, MDA-MB-231 and MDA-MB-468 (Fig. 5d). *DDAH1* gene expression was upregulated in all BC lines except MCF-7, and *DDAH2* was upregulated in MCF-7, BT-474, MDA-MB-231 and MDA-MB-468 cells (Fig. 5g and h).

## Discussion

The present study shows that metabolism of the semi-essential, proteinogenic amino acid L-arginine may be of clinical importance in breast cancer. Its concentrations within representative breast cancer cell lines vary considerably according to molecular breast cancer subtypes. These differences in L-arginine-related metabolite concentrations are related to gene expression profiles of L-arginine-metabolizing enzymes within breast cancer subtypes. In addition, plasma concentrations of L-arginine and its metabolites in plasma of patients with primary breast cancer mirror the principal differences observed between cell lines. The L-arginine-derived metabolites ADMA and L-citrulline are significantly and prospectively associated with recurrent disease and total mortality in patients with luminal A breast cancer and triple-negative breast cancer, respectively.

The group of breast cancer patients that we analyzed was a representative cohort of 243 women aged 50–70 years with primary breast cancer. The distribution of histological subtypes, tumor staging, hormone receptor status, allocation to clinical subtypes of breast cancer and to treatment strategies was highly comparable to breast cancer cohorts previously described by other investigators [5]. Whilst most L-arginine-related metabolite concentrations as measured in the systemic circulation were comparable to matched healthy controls, there were a few noteworthy observations in this cohort. L-arginine concentration in the total cohort of breast cancer patients was significantly lower and the L-citrulline/L-arginine ratio was significantly higher as compared to healthy controls. In linear regression analyses, all L-arginine-related metabolites showed significant positive or negative (L-arginine) correlation with age. We had previously reported L-arginine, ADMA, and SDMA concentrations in healthy reference cohorts to show an age-dependent decrease (L-arginine; [23] or increase (ADMA, SDMA; [24–26]); therefore, we corrected these analyses in our present study for age as a covariate. Within the breast cancer patient group, non-survivors and patients with recurrent disease had significantly higher mean L-arginine levels than survivors and patients without recurrent disease, respectively. Non-survivors also had a significantly higher mean ADMA concentration than survivors.

The results of our analysis in representative breast cancer cell lines suggest that high L-arginine concentrations may have been driven primarily by ER-positive breast cancers, as both MCF-7 and BT-474 cells showed significantly and greatly elevated intracellular L-arginine levels. By contrast, elevation of the L-citrulline/L-arginine ratio appears to be mainly promoted by triple-negative breast cancers, as MDA-MB-468 cells showed significantly elevated intracellular L-citrulline/L-arginine ratio. It is remarkable that these intracellular differences in metabolite concentrations and ratios closely resemble those measured in plasma in the patient cohort, i.e. extracellular metabolite levels. We did target intracellular concentrations in breast cancer cell lysates in-vitro in order to directly assess intracellular differences in L-arginine metabolism within the tumor cells, an analysis that is hardly feasible in patients.

Metabolite concentrations, whether measured in plasma in-vivo or intracellularly in-vitro, need to be interpreted with caution, as all of the L-arginine-related metabolites are subject to more than one enzymatic pathway and, thus, underlie complex metabolic regulation. Nonetheless, nitric oxide synthase catalyzes the conversion of L-arginine to NO and L-citrulline, thus leading to lower L-arginine and higher L-citrulline concentrations. Based upon this reasoning, this ratio has been suggested to be a surrogate marker of total NO synthesis activity [27]. Elevation of the L-citrulline/L-arginine ratio in MDA-MB-468 cells is thus suggestive of higher NO synthesis activity; this is in line with reports of upregulated activity of inducible NO synthase in triple-negative breast cancer and the induction of a basal-like transcription pattern in ER-negative patients [28–30]. Our present data suggest that this may relate to posttranscriptional regulation at the protein and/or activity levels, as *NOS II* mRNA expression levels in MDA-MB-468 cells were very low. In addition, our clinical observation that L-citrulline concentrations in plasma are positively and prospectively associated with recurrent disease and total mortality of patients with triple-negative breast cancer also support this hypothesis. A similar reasoning may relate to L-ornithine as a product of arginase and the L-ornithine/L-arginine ratio as a surrogate marker of overall arginase activity. MDA-MB-468 cells were prominent with high levels of this L-arginine metabolite; however, we could not confirm importance of L-ornithine for clinical outcome in the patient cohort. Arginase-2 metabolizes L-arginine into L-ornithine, which is then further processed, amongst others, into polyamines. The latter molecules have been shown to be cell cycle regulators [31, 32]; thus, high proliferative activity of triple negative cells might relate to polyamine-driven enhancement of cell proliferation [33, 34]. In previous studies, high *ARG2* expression was linked to worse metastasis-free and overall survival in patients with primary breast cancer [35]. In addition, knockdown of *ARG2* in cultured triple-negative breast cancer cells markedly reduced cell growth [36]. These data suggest an important role of L-arginine metabolism by arginase-2 for proliferation and outcome of some – if not all – breast cancer subtypes.

By contrast to MDA-MB-468 cells, the high elevation of L-citrulline and L-ornithine in BT-474 cells was related to high L-arginine concentration in this cell line; it is therefore suggestive of a lack of regulation of the NO synthesis and arginase pathways in this cell type. Interestingly, the two triple-negative cell lines, MDA-MB-231 and MDA-MB-468 showed major differences in gene expression and metabolite concentrations, suggesting that differences in L-arginine metabolism may contribute to biological differences between the basal-like and the claudin-low subtypes of triple-negative breast cancer [37].

The dimethylarginines, ADMA and SDMA, are the major endogenous end products of protein Larginine methylation in humans. The PRMT enzymes are classified into three groups; type 1 PRMTs (comprising PRMTs 1, 2, 3, 4, 6, and 8) mediate asymmetric dimethylation of proteins which results in ADMA release during proteolytic protein breakdown, type 2 PRMTs mediate symmetric dimethylation (i.e., SDMA formation; PRMTs 5 and 9), and type 3 PRMTs solely catalyze monomethylation of proteins (PRMT7) (for review of PRMTs, cf. [38]), the type 2 PRMTs 5 and 9 to be downregulated in most BC cell lines, and PRMT7 to be downregulated in the two triple negative BC cell lines. We did not find strict correlations between these differential mRNA expression patterns and intracellular ADMA and SDMA metabolite concentrations in the cell lines. However, the ADMA-degrading enzymes, *DDAH1* and *DDAH2* were both upregulated in almost all BC cell lines as compared to the MCF-12A normal breast epithelial cell line. Taken together, this data suggests that asymmetric protein L-arginine methylation may be a differentially upregulated process in BC tumor biology; BC cells protect themselves from the potentially cytotoxic effects of dimethylarginines by upregulating their DDAH-mediated breakdown to L-citrulline and dimethylamine. The involvement of DDAH1 in ADMA metabolism has been demonstrated clearly, whereas the role of DDAH2 has been debated for long due to controversial results; recent data suggest that DDAH2 may not contribute to ADMA metabolism [39]. In addition, previous data showed that *DDAH1* is overexpressed in some triple-negative breast cancer cell lines, contributing to their aggressiveness [40]. Asymmetric protein dimethylation has previously been shown to target important cellular processes like regulation of gene transcription, pre-mRNA splicing, DNA damage and immune signaling [41]. These processes drive cell proliferation, cell invasion and metastatic ability in breast cancer cells [42]. For example, knockdown of *PRMT1* in MDA-MB-468 cells was shown to reduce EGF receptor-mediated signaling [43]. Thus, PRMTs have been proposed as potential new targets for therapy in breast cancer and other tumor entities (for Reviews, cf [44–46]).

Our present clinical data support a major role of L-arginine-related molecular pathways in breast cancer outcome. High ADMA concentrations in plasma may result from upregulated type 1 protein L-arginine demethylation with resulting spillover of ADMA into plasma. High ADMA was associated with high rates of tumor recurrence and total mortality, specifically in women with ER-positive breast cancer. This finding, if confirmed in larger clinical studies, may confer a novel opportunity for individualized treatment beyond ER antagonists in ER-positive breast cancer. However, studies in patient cohorts with non-malignant diseases as well as in population-based cohorts have also shown that high ADMA concentration is a predictor of total mortality and of cardiovascular events [47–49]. Decreased plasma L-arginine and elevated ADMA have been shown to be associated with cardiovascular side effects during doxorubicin therapy of breast cancer [50]. We measured plasma L-arginine metabolites before initiation of cancer therapy in our study. Thus, future prospective studies should highlight causes of death in patients with high ADMA concentration and clearly define the time of blood sampling as related to anticancer therapy.

Considering the multi-faceted implications of L-arginine metabolism in cancer and the crucial role of L-arginine availability for cell growth, L-arginine deprivation therapy has been discussed as a potential strategy for tumor regression in breast cancer and in other tumor entities [51, 52]. Hu and co-workers studied L-arginine metabolites in a small group of Chinese breast cancer patients [53]; however, L-arginine concentrations in that study were extremely low as compared to published reference ranges [23]. Our present data showing the high degree of variability of expression and metabolite concentrations of the different L-arginine-metabolizing pathways suggest that such a strategy should include determination of the expression levels of L-arginine-metabolizing pathways and their functional importance to optimize the individual response rates to such deprivation strategies. This complexity is stressed, for example, by a study by Cao and colleagues who showed that L-arginine supplementation may inhibit the growth of breast cancer [16]. This may be caused by secondary activation of the pro-survival autophagic response during L-arginine starvation, as shown in ovarian cancer cells [54]. Currently, L-arginine remains a two-faced molecule in cancer biology and cancer therapy [55].

Our study is limited by its relatively small number of breast cancer patients included from a single center, limiting the power to significantly detect minor differences in L-arginine-metabolizing pathways between subgroups. Its strength is that all blood samples had been drawn before the initiation of cancer treatment, and all patients had been treated according to current guidelines; nonetheless, an influence of differences in systemic therapy on outcome cannot be ruled out. We have previously validated our analytical method for quantitation of L-arginine and its metabolites, including long-term storage [56]. Nonetheless, our study is limited by the fact that we were unable to fully match breast cancer patients with healthy controls due to the unavailability of healthy blood donors of advanced age. However, another strength of our study lies in the combination of prospective clinical outcome data, state-of-the-art analytical methods for metabolite quantification, and mRNA expression analyses in representative cell lines in-vitro.

In summary, major differences in gene expression of L-arginine-metabolic enzymes as well as intracellular L-arginine-related metabolite concentrations can be detected in breast cancer cell lines representing different molecular subtypes. Some, but not all of these differences are mirrored in plasma of patients with breast cancer, in whom such differences in metabolite concentrations are associated with outcome. This data supports the hypothesis that L-arginine metabolism is an important determinant of the biological activity of different breast cancer subtypes. Further studies, both clinical and experimental, are required to define the roles of the various L-arginine-metabolizing pathways as potential therapeutic targets for breast cancer therapy.

## Electronic supplementary material

Below is the link to the electronic supplementary material.

Supplementary Material 1

## Data Availability

The data that support the findings of this study are not openly available due to reasons of sensitivity and are available from the corresponding author upon reasonable request. Data are located in controlled access data storage at University Medical Center Hamburg-Eppendorf.
