# Supplementary material for: L-arginine dependence of breast cancer – molecular subtypes matter
Source: BMC Cancer. 2025 Mar 26;25:546. doi: 10.1186/s12885-025-13908-4 (PMC11948839; doi:10.1186/s12885-025-13908-4)
Supplement: Supplementary file 1 — Supplementary Material 1 [file 12885_2025_13908_MOESM1_ESM.pdf]

L-Arginine dependence of breast cancer –

molecular subtypes matter.

Juliane Hannemann[1]*, Leticia Oliveira-Ferrer[2], Anne Kathrin Goele[1], Yoana Mileva[1], Fiona Kleinsang[1], Antonia Röglin[1], Isabell Witzel[3], Volkmar Müller[2], Rainer Böger[1]

[1] Institute of Clinical Pharmacology and Toxicology and [2]Department of Gynecology, University Medical Center Hamburg-Eppendorf, Hamburg, Germany, [3]Department of Gynecology, University Hospital Zürich, Zürich, Switzerland

Supplementary Material

Supplementary Table

Supplementary Table 1. Specific TaqMan gene expression assays for real-time quantitative PCR for each of the genes analyzed in this study.

Target Gene	Taqman gene expression assay identifier
ARG1	Hs00968979_m1
ARG2	Hs00982833_m1
DDAH1	Hs00201707_m1
DDAH2	Hs00967863_g1
ASS1	Hs01597989_g1
ASL	Hs00902699_m1
PRMT1	Hs01587651_g1
PRMT2	Hs00895397_m1
PRMT3	Hs00969596_m1
PRMT4	Hs00406354_m1
PRMT5	Hs01047356_m1
PRMT6	Hs05054640_s1
PRMT7	Hs00219300_m1
PRMT8	Hs00998598_m1
PRMT9	Hs00378858_m1

Gene names: ARG, arginase; DDAH, dimethylarginine dimethylaminohydrolase; ASS, L-arginine succinate synthase; ASL, L-arginine succinate lyase; PRMT, protein-arginine methyltransferase.

Supplementary Figures

Supplementary Figure 1.

Schematic representation of major L-arginine-metabolizing pathways. L-arginine is converted to L-citrulline and nitric oxide by nitric oxide synthases or to L-ornithine and urea by arginases. Further, it can be methylated by a family of protein-arginine N-methyltransferases (PRMTs), of which three distinct subtypes can be discriminated. The dimethylarginines, ADMA and SDMA, are released upon hydrolytic degradation of methylated proteins. ADMA is metabolically cleaved to L-citrulline and dimethylamine by dimethylarginine dimethylaminohydrolases (DDAHs).

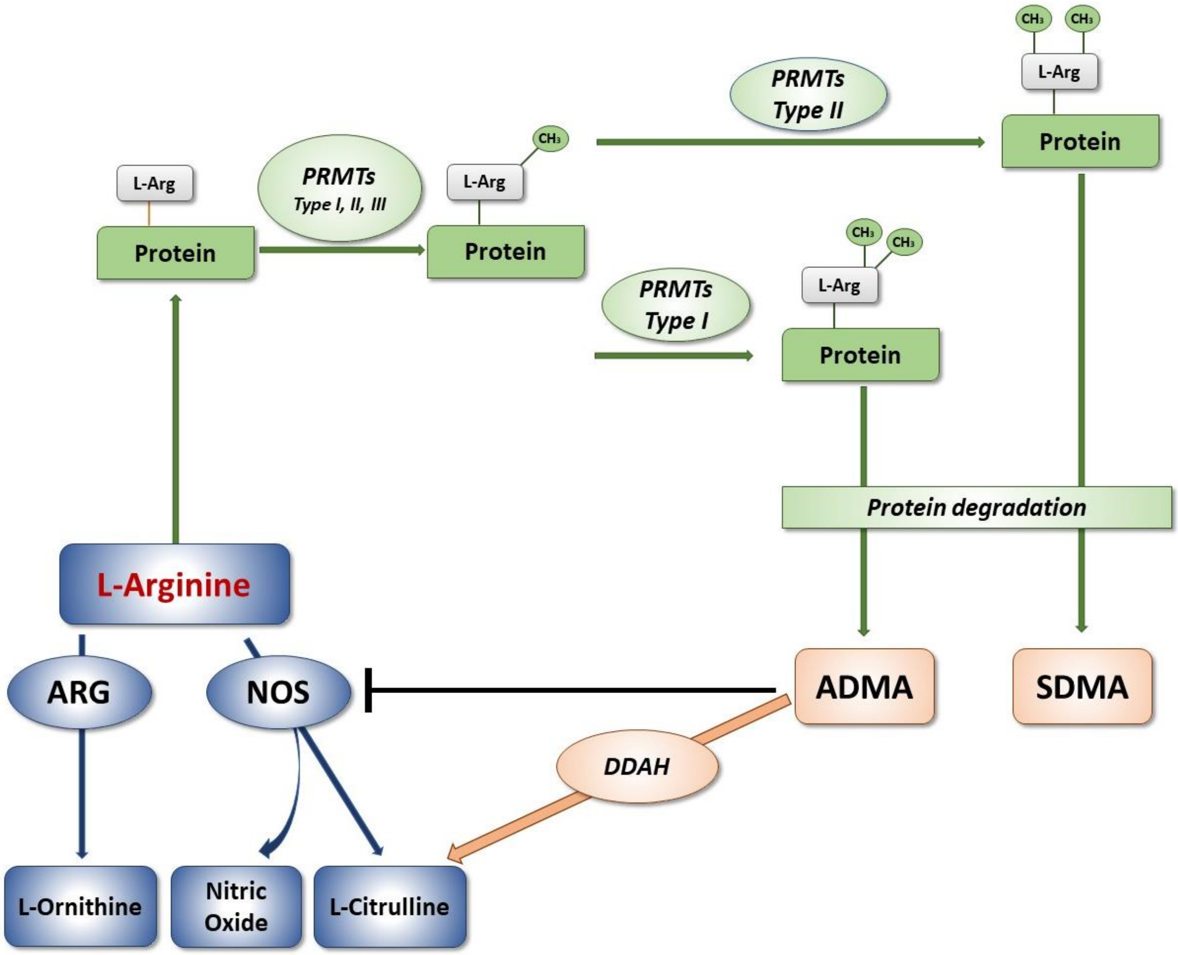

Supplementary Figure 2.

CONSORT flow diagram of the breast cancer study population.

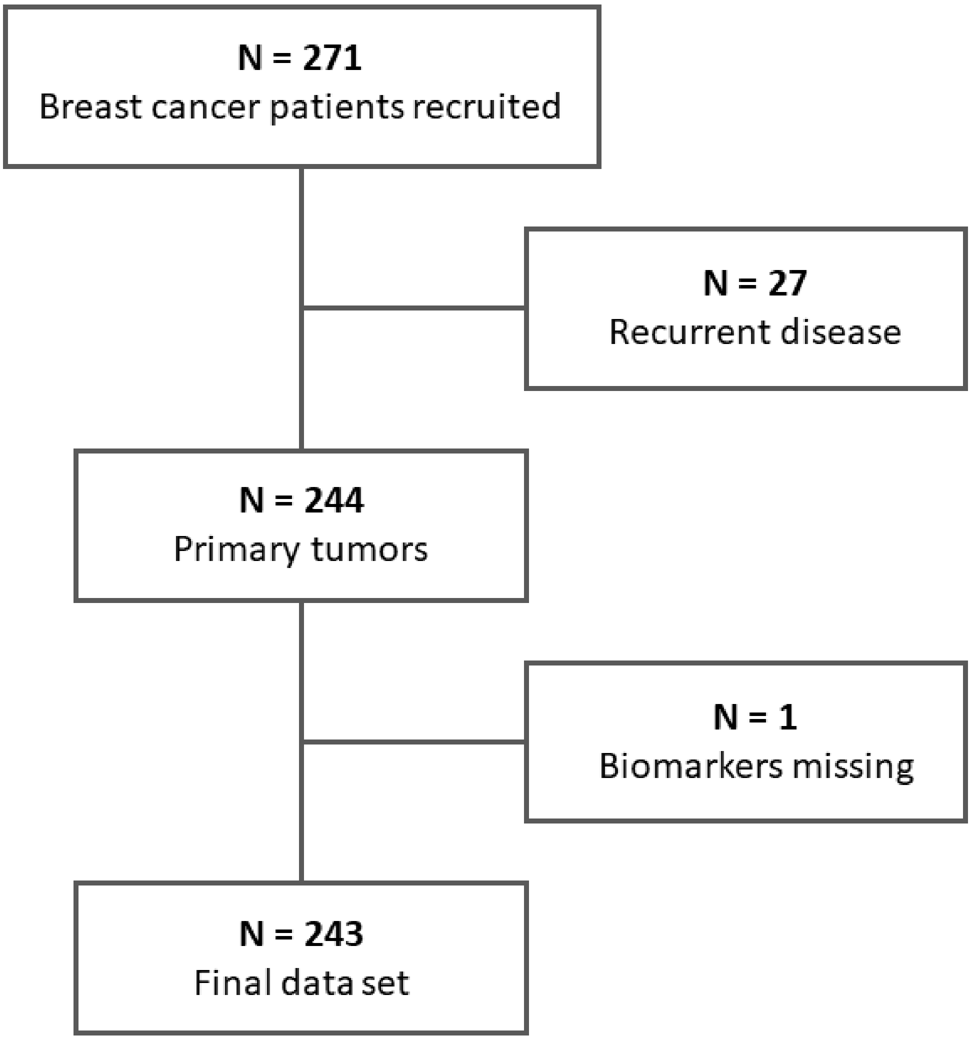

Supplementary Figure 3.

Gene expression measured by quantitative real-time RT-qPCR for genes involved in the L-arginine salvage pathway: a) argininosccinate synthase-1 (ASS1), b) argininosuccinate lyase (ASL). ** p < 0.001 as compared to MCF-12A normal breast epithelial cells in two-way ANOVA followed by Dunnett's multiple comparisons test.

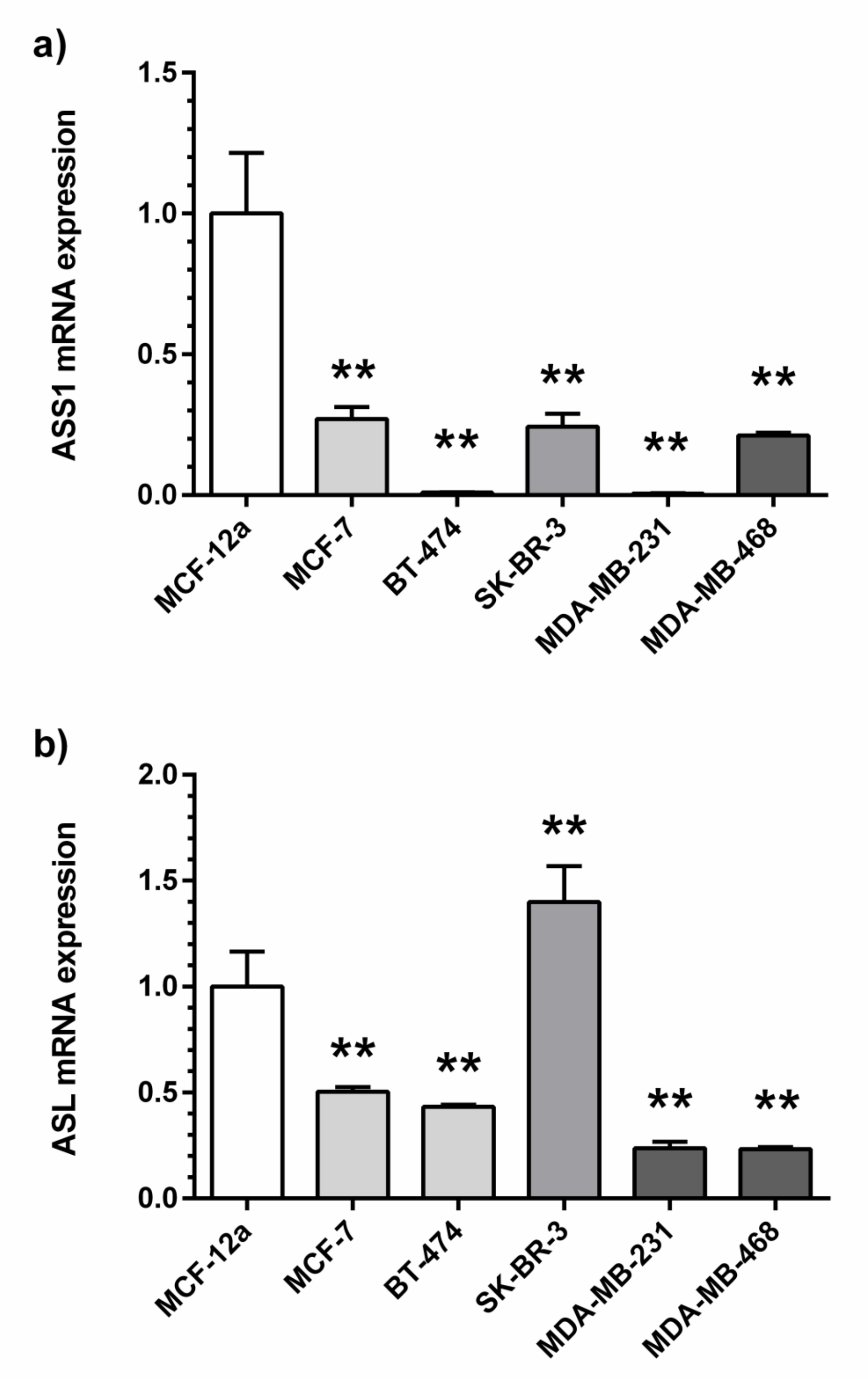

Data documentation for study participant No. ___________________

Date of documentation: ______________________

General Patient Characteristics

Date of birth	____________________	Date of diagnosis	____________________
Body weight at diagnosis	_______________ (kg)	Height	_______________ (cm)
Date of surgery	____________________	Serum creatinine (pre-surgery)	____________ (mg/dL)

Diagnosis / Medical History

☐ Primary breast cancer ☐ Recurrent carcinoma ☒ left ☐ right breast

☐ IDC (Invasive, ductal carcinoma)

☐ ILC (Invasive, lobular carcinoma)

☐ DCIS (Ductal carcinoma *in situ*)

☐ LCIS (Lobular carcinoma *in situ*)

Remarks:
(e.g., IDC with DCIS components; bifocal; microcalcifications)
____
____
____

Histological Type

☐ ductal	☐ lobular	☐ papillary	☐ serous
☐ endometrioid	☐ mucinous	☐ serous-papillary	☐ clear-cell
☐ tubular	☐ inflammatory	☐ other	☐ not specified

Previous Malignant Disease(s)

☐ No

☐ Yes

If yes, which (type, date, etc.) ____

____

____

Family History of Breast Cancer or other Malignant Diseases

____

____

____

TNM-State & Grading

Grading: ☐ 1 ☐ 2 ☐ 3

Tumor size	p**T** ____________		
Sentinel node	**N** ____________	positive LN/dissected LN	____/____
Metastasis	**M** ____________	If positive, where?	_______________
Lymphatic invasion	☐ L0	☐ L1	
Vascular invasion	☐ V0	☐ V1	☐ V2
Resection	☐ R0	☐ R1	☐ R2
Date of secondary resection	_____________	Thereafter R0?	☐ yes ☐ no

Receptor State / Molecular Markers

ER	_____ /12	☐ N/A		PR	_____ /12	☐ N/A
HER2	☐ 0 (neg)	☐ 1 (pos)	☐ N/A	Ki-67	_____ %	☐ N/A
E-cadherin	☐ neg	☐ pos	☐ N/A			

Therapy

Surgery

☐ Mastectomy ☐ Lumpectomy ☐ ________________ _____ ☐ None

Chemotherapy

☐ None ☐ Yes, regimen: ____
____

Radiotherapy ☐ Yes ☐ No

Endocrine Therapy

☐ None ☐ Yes, which: ____
____

Follow Up

Phone call on: ____________________________________(date)

Talked with: __________________________________

Deceased ☐ No ☐ Yes, on ________________________(date)

Cause of death: ____

Recurrence ☐ No ☐ Yes, diagnosed on ______________(date)

2nd malignancy ☐ No ☐ Yes, diagnosed on ______________(date)

Which: _________________________

Remarks:

____

____

____

____

____

____

____

____

____

____

____

____
